# Digital Twin of a Gear Root Crack Prognosis

**DOI:** 10.3390/s23249883

**Published:** 2023-12-17

**Authors:** Omri Matania, Eric Bechhoefer, Jacob Bortman

**Affiliations:** 1 BGU-PHM Laboratory, Department of Mechanical Engineering, Ben-Gurion University of the Negev, P.O. Box 653, Beer Sheva 8410501, Israel; omrimatania@gmail.com; 2 GPMS International Inc., 93 Pilgram Place, Waterbury, VT 05676, USA; eric@gpms-vt.com

**Keywords:** digital twin, gear, root crack, remaining time until maintenance, prognosis

## Abstract

Digital twins play a significant role in Industry 4.0, offering the potential to revolutionize machinery maintenance. In this paper, we introduce a new digital twin designed to address the open problem of predicting gear root crack propagation. This digital twin uses signal processing and model fitting to continuously monitor the condition of the root crack and successfully estimate the remaining time until immediate maintenance is required for the physical asset. The functionality of this new digital twin is demonstrated through the experimental data obtained from a planetary gear, where comparisons are made between the actual and estimated severity of the fault, as well as the remaining time until maintenance. It is shown that the digital twin addresses the open problem of predicting gear root crack propagation.

## 1. Introduction

Digital twins are one of the most significant advancements in Industry 4.0 [1,2]. At their core, digital twins monitor the condition of physical assets through real-time data collected by sensors integrated into the assets [3,4]. They enable tracking the status of these assets and, furthermore, forecasting their future behavior based on optional examined scenarios, thereby facilitating informed decision making regarding their usage [5,6]. Digital twins are applied for various tasks [7,8,9,10].

Rotating machines are valuable physical assets that, in most cases, demand intricate maintenance [11,12]. In addition to the necessity of detecting faults in these machines, there is a need to predict future maintenance schedules to enhance availability and prevent unexpected downtime, which can incur substantial financial costs [13]. For instance, in the case of helicopters, a fault detected too late may lead to the grounding of the aircraft, affecting fleet availability and causing significant financial losses.

Currently, for critical rotating machines such as helicopters, there are well-established techniques for fault detection and classification [14,15]. According to these techniques, the signal is angular resampled [16,17] from the time domain to the cycle domain, and then, the monitored component is isolated using methods such as synchronous averaging in the case of gears [18,19]. Based on the isolated signals, the features are extracted to detect a fault and classify it as a fault in the gear [20,21,22]. However, the current techniques do not enable the estimation of fault severity, and the remaining time until immediate maintenance action should be taken. While various machine learning approaches exist for these tasks, they are not applicable to critical rotating machines such as helicopters, where faulty data are not available during the training phase [23].

One of the potential faults in rotating machines is a crack in the root of one of the gearbox wheels, a similar fault pattern of which has already led to several accidents in the past [24,25]. This article introduces a new digital twin for monitoring this fault based on the fusion of four vibration measurements, enabling the tracking of the fault’s severity and estimation of the time remaining until immediate maintenance is required. The capabilities of the new digital twin are demonstrated using the HUMS 2023 benchmark dataset [26].

This article consists of five sections. Section 2 provides a description of the theoretical background, while Section 3 presents the tested dataset, HUMS 2023 benchmark. In Section 4, the new digital twin is introduced and demonstrated, and in Section 5, this article is summarized.

## 2. Theoretical Background

In this section, this paper’s theoretical background is presented. First, in Section 2.1, a background about condition-based maintenance of critical rotating machines is given. In Section 2.2, the theoretical foundation for analyzing vibrations in rotating components is provided. Then, in Section 2.3, Paris’ law is explained. It is used to model the crack growth in the new digital twin. Lastly, in Section 2.4, an explanation about digital twinning is given.

### 2.1. Condition-Based Maintenance of Critical Rotating Machines

Complex mechanical systems such as helicopters, trains, and wind turbines require expensive maintenance to prevent accidents that can cost human lives or cause severe damage to the system itself [27,28]. The maintenance cost of these systems over their operational lifespan can often be much higher than the initial cost of the system. For example, the purchase price of a Bell 407GX helicopter can range approximately from USD 3 million to USD 4 million. The maintenance cost per flight hour can be estimated at approximately USD 1200, which becomes more expensive as the helicopter gets older. Assuming 400 flight hours per year, this results in an annual cost of approximately USD 0.5 million. Over an optional operational lifespan of 30 years, with several different operators, the maintenance costs can add up to USD 15 million or even more. This significantly exceeds the purchase price, being four to five times as much.

Complex mechanical systems are maintained through preventive maintenance, where inspections and replacements of various components are scheduled based on a manufacturer’s predetermined schedule, which is established based on failure statistics and known maintenance principles [29]. This type of maintenance presents three main issues: (1) the maintenance costs are high because many checks and component replacements are carried out under perfectly healthy conditions, (2) maintenance actions themselves can introduce failures, and (3) the misdetection of failures that can lead to catastrophic accidents or system unavailability [29].

In recent decades, condition-based maintenance methods have been developed to enhance maintenance practices [27,29,30,31]. One common approach to condition-based maintenance in complex rotating machinery is the use of vibrations analysis [14,15,32]. In this approach, vibration and shaft speed sensors are installed on the rotating parts of the system (e.g., a helicopter’s rotor, gear casing, etc.), signal processing algorithms are employed to detect faults and identify their sources [22,33].

### 2.2. Vibration Analysis

In this paper, two well-known algorithms are utilized to analyze the vibration signal: angular resampling [16,34] and synchronous average [19,35]. A profound understanding of the component’s physical behavior guides signal processing algorithms tailored uniquely for each rotating component type.

**Figure 1:**
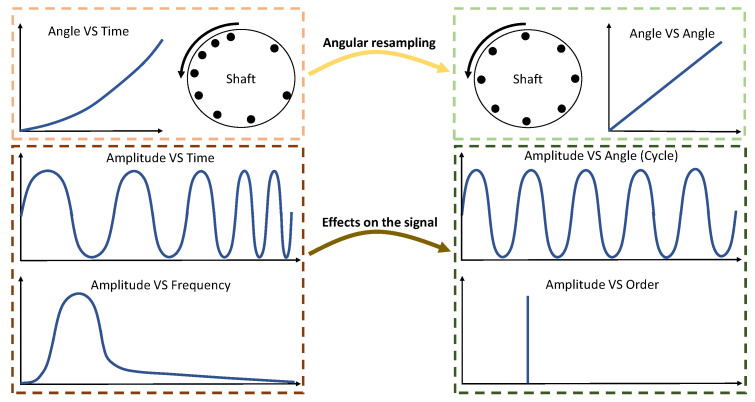
An illustration of angular resampling. Reproduced from Ref. [23] based on Ref. [17].

In many cases, vibration analysis is conducted in the frequency domain because of the periodic nature of the vibrations. In practice, the periodicity of the signals is related to the phase of the rotating axis rather than time since the rotational speed is not constant. To address this challenge, angular resampling can be employed [16,34]. As depicted in Figure 1, in the cycle domain, the signal becomes periodic after angular resampling. In this algorithm, the phase of the shaft is calculated based on the rotating speed, and then, the signal is resampled according to new times with constant phase intervals. The frequency domain corresponding to the cycle domain is referred to as the order domain.

**Figure 2:**
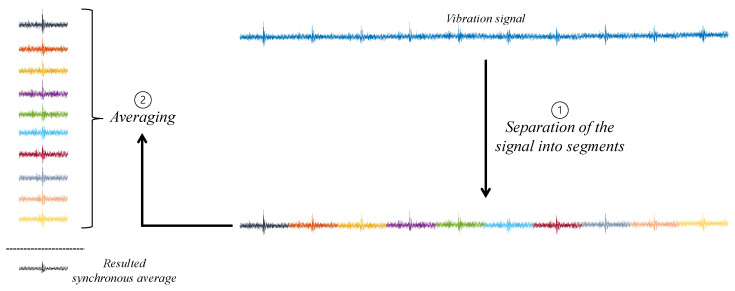
Diagram of synchronous average algorithm.

Synchronous averaging is a crucial step in gear diagnosis, serving two primary purposes: noise reduction and the damping of vibrations from other rotating components [36]. This algorithm allows the isolation of gear vibrations and is relatively straightforward to implement, as demonstrated in Figure 2. The vibration signal is divided into consecutive segments, and these segments are subsequently averaged [19,35].

The objective of synchronous averaging is to isolate the vibrations of the specific component under consideration. This is achieved by enhancing the signal-to-noise ratio, thereby minimizing interferences from other rotating components [19,37] and random noise [38]. The reduction in random noise can be analyzed using Equations (1) and (2). The synchronous average is computed using Equation (1), where sa represents the calculated synchronous average with N samples, M is the number of averaged segments, and sig is the signal in the cycle domain with M·N samples. Assuming that the random noise is independently identically distributed, it can be deduced that the original variance of each sample is reduced from $\sigma^{2}$ to $\frac{\sigma^{2}}{M}$, as illustrated in Equation (2).

(1) $$sa\left[n\right]=\frac{1}{M}\sum_{m=0}^{M-1}sig\left[m\cdot N+n\right]$$

(2) $$Var\left(sa\left[n\right]\right)=Var\left(\frac{1}{M}\sum_{m=0}^{M-1}sig\left[m\cdot N+n\right]\right)=\frac{1}{M^{2}}\sum_{m=0}^{M-1}Var\left(sig\left[m\cdot N+n\right]\right)=\frac{1}{M^{2}}\cdot M\cdot \sigma^{2}=\frac{\sigma^{2}}{M}$$

**Figure 3:**
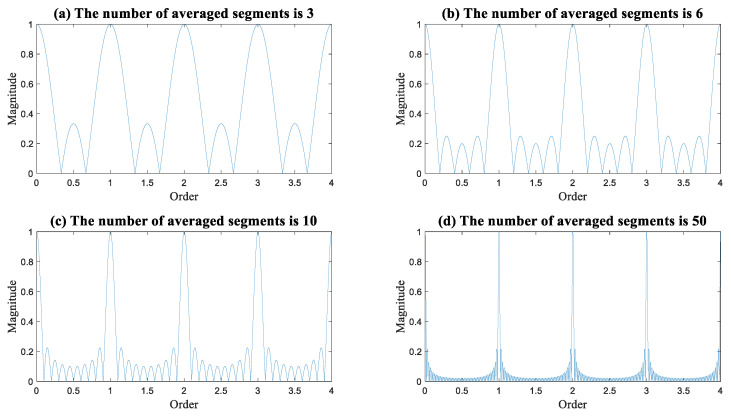
The generated filter through synchronous averaging concerning the sampled original infinite continuous signal.

The analysis of reducing interference signals has been conducted in various studies, such as Refs. [18,19]. Regarding the sampled infinite continuous signal, synchronous averaging is a filter that isolates the complete orders, as illustrated in Figure 3. With an increase in the number of averaged segments, the filter becomes more selective [39].

### 2.3. Paris’ Law

Paris’ law describes crack size growth [40,41]. The rate of growth can be described using Equation (3).

(3) $$\frac{da}{dN}=C{\left(∆k\right)}^{m}=C{\left(\sigma \cdot \alpha \cdot \sqrt{\pi \cdot a}\right)}^{m}=C{\left(\sigma \cdot \alpha \cdot \sqrt{\pi }\right)}^{m}\cdot a^{\frac{m}{2}}$$

where ∆k is stress intensity factor, σ is the delta stress, α is a correction factor due to the shape of the component, D is the material constant, N is the number of loading cycles, m is the crack growth exponent, and a is the crack size. When the initial crack size is known, the differential equation describing Paris’ law enables relating between the number of loading cycles and the crack size.

### 2.4. Digital Twins

Digital twining has been defined in various ways, such as a virtual organization, a representation in virtual reality, high-fidelity simulation, and an emulation facility [1]. Digital twins represent a groundbreaking concept that has achieved significant attention in various fields, including manufacturing, healthcare, urban planning, and more. A digital twin is a virtual counterpart of a physical object, system, or process. It can be utilized for various applications, such as predictive maintenance, performance optimization, and decision support [3,4,42,43,44,45,46,47,48,49,50].

The concept of a digital twin has its origins in computer-aided design and computer-aided engineering technologies, which have been employed for decades to model and simulate physical systems. Nevertheless, the term “digital twin” gained prominence with the advent of cyber-physical systems. Some people attribute the first official use of this concept to 2002. Over the years, digital twin technology has extended to various industries, propelled by advancements in sensors, data analytics, and computational power [1,3,4]. Leading manufacturing companies, such as Ford Motor Company and Volvo Group Global, have implemented the digital twin concept to enhance performance, evaluate and optimize designs, and validate changes [1].

For instance, General Electric developed digital twins for aircraft engines, Ford Motor Company improved assembly line performance by leveraging digital twins for design evaluation, while Volvo Group Global demonstrated the validation of changes using a digital twin. Currently, major software vendors support the development of virtual factories through integrated solutions for product, process, and system design [1].

**Figure 4:**
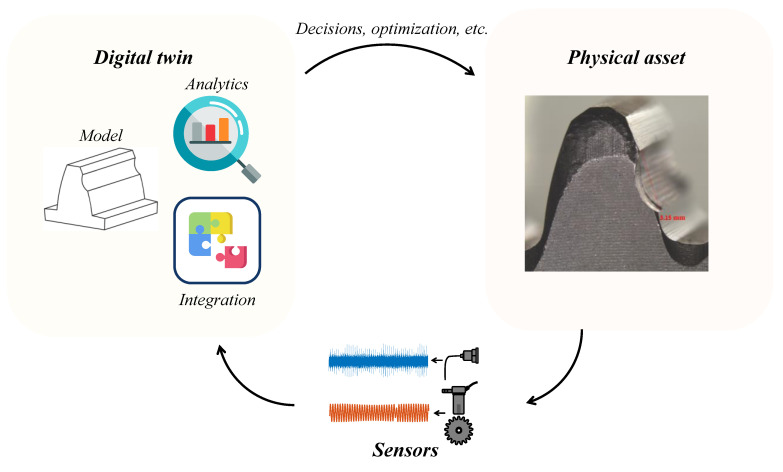
The digital twin is a component of a larger cycle that includes the physical asset, sensors, and the digital twin itself.

A digital twin is one component of a larger cycle, as depicted in Figure 4: (1) the physical asset, which is the real-world entity represented by the digital twin; (2) sensors and data sources capture real-time information about the physical asset; (3) the digital twin that represents the physical asset and can be continuously updated to mirror the real-world state. The digital twin involves data processing and integration from various sources to ensure that the virtual model accurately reflects the physical object’s condition. It also incorporates analytics and algorithms for analyzing and interpreting sensor data, extracting insights, and making predictions about the physical system’s behavior.

In the context of this study, the digital twin assists in simplifying a complex problem: monitoring and predicting the health status of a rotating component. It achieves this by continuously tracking the current state and predicting the future development of the crack for that specific state instead of trying to solve a challenging problem by solely relying on current measurements to understand the machine’s state and then evaluating all the potential ways the crack could progress.

## 3. Experimental Dataset

**Figure 5:**
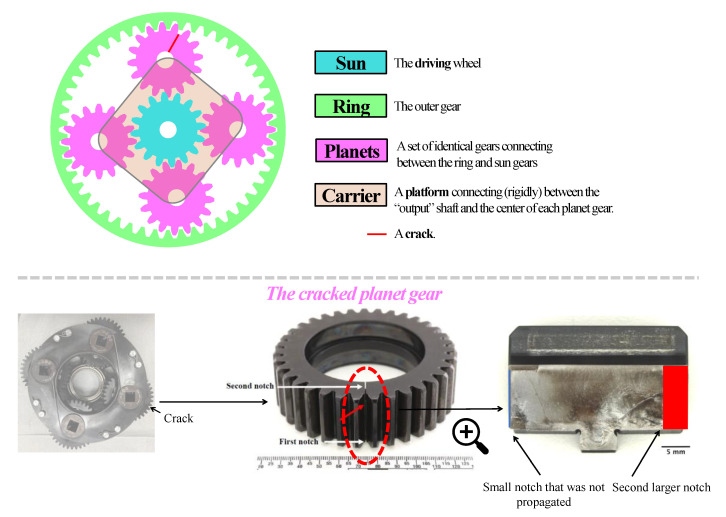
An explanation of the components of planetary gear and the faulty gear of HUMS 2023 benchmark dataset. The cracked area is indicated by a red arrow and a red dotted circle. More information is available in Ref. [26].

The HUMS 2023 benchmark dataset comprises a total of 4 × 526 data records of a planetary gear crack collected over a seven-day period, with 526 records allocated to each of the four vibration channels. The shaft speed was measured using a tachometer connected to the National Instruments PXI-6259 system, which measures the revolutions per second. Interested readers can find detailed information about this dataset in Ref. [26]. An explanation about the components of planetary gear and the gear with the crack are depicted in Figure 5. The sun, planets, and ring gears had 27, 35, and 99 teeth, respectively.

**Figure 6:**
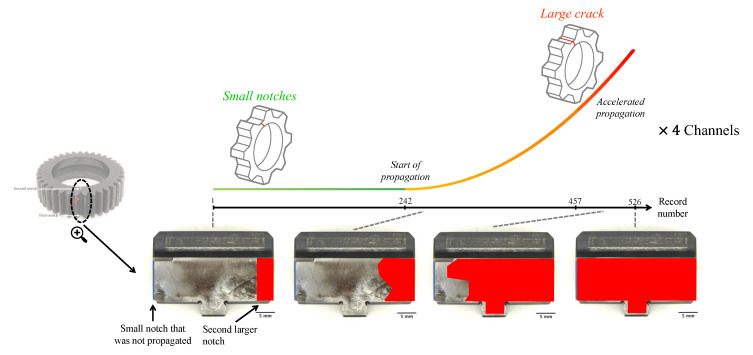
Crack propagation of HUMS 2023 benchmark dataset. The red area represents the propagation of the crack along the experiments.

The experiment took place at the helicopter transmission test facility in Australia’s Defense Science and Technology Group [51]. The benchmark dataset was generated from a propagating fatigue crack in a planet gear within a helicopter’s main rotor gearbox, as depicted in Figure 6. The cracked planet gear, as shown in Figure 5, features two notches, one on each side. Initially, prior to the commencement of the 4 × 526 vibration records, the first (smaller) notch did not induce crack propagation. Subsequently, the gearbox was disassembled, and a second (larger) notch was introduced on the opposite side. It successfully resulted in the propagation of a fatigue crack from the second notch, as illustrated by the trend in Figure 6.

After the conclusion of the experiment, the gear underwent fractography analysis to assess the fault propagation over time. The estimation of the start of crack propagation was recorded at 242, and the commencement of accelerated propagation was estimated at record 457, as depicted in Figure 6.

**Figure 7:**
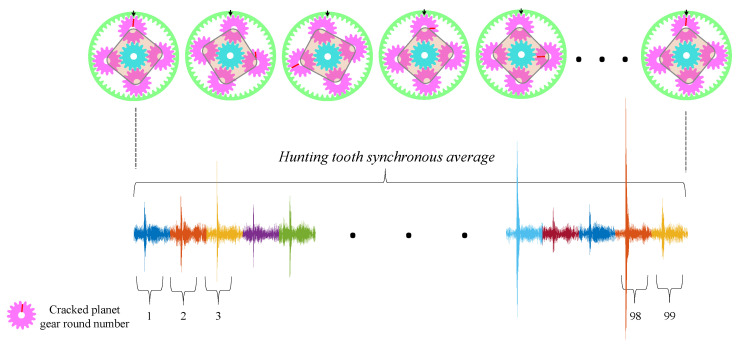
Illustration of hunting tooth synchronous average.

As illustrated in Figure 7, each record from the 4 × 526 vibration records represents a synchronous average corresponding to the interaction of the crack region with the ring, i.e., 99 (the number of teeth on the ring) complete revolutions of the cracked planet gear, each revolution contains 4096 samples. This synchronous average is designated as the hunting tooth synchronous average. At the end of the hunting tooth synchronous average, the same tooth of the cracked gear contacts the exact tooth on the ring, just as in the beginning of the record. It should be noted that the cracked planet gear revolves around the center of the ring much faster than the rotation speed of the hunting tooth; this occurs just after 99/35 planet gear rotations compared to 99 rotations of the gear to complete one hunting tooth rotation.

## 4. The New Digital Twin

**Figure 8:**
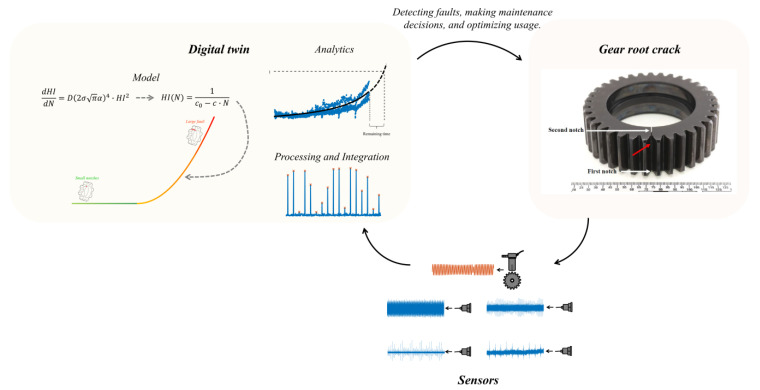
General block diagram of the new digital twin.

As shown in Figure 8, the new digital twin is a component of a larger cycle that includes four vibration sensors and one speed sensor, along with the physical asset, the gear. The new digital twin comprises two main stages: (1) signal processing for analyzing the measured data and (2) data analysis for estimating the severity of the crack and predicting the remaining time until immediate maintenance action should be taken. The severity and remaining time estimations are based on the integration of the analyzed data and the crack propagation model based on Paris’ law described in Section 2.3. Section 4.1 describes the signal processing stages, while Section 4.2 covers the severity and remaining time estimations. Section 4.3 demonstrates the new digital twin using the HUMS 2023 benchmark dataset described in Section 3.

### 4.1. Signal Processing Stage

**Figure 9:**
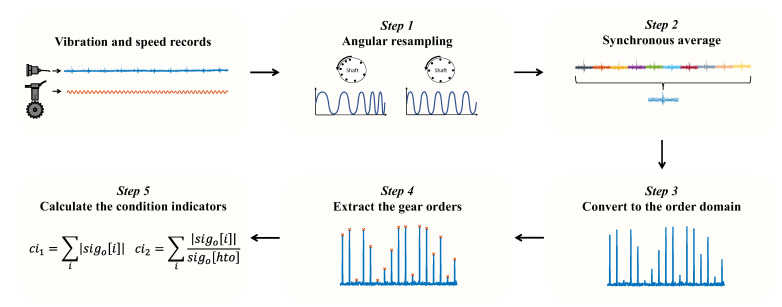
The signal processing stage of the new digital twin.

The signal processing of the vibration signals is repeated for each channel separately, as illustrated in Figure 9. The signal processing stage comprises the following steps:

1. The signal is angularly resampled from the time domain to the cycle domain using the speed record [16,34].
2. The synchronous average corresponding to the hunting tooth is calculated [19,35].
3. The signal is converted to the order domain (analogous to the frequency domain) using discrete Fourier transform.
4. The orders of the gear are extracted, and these orders are used to calculate the condition indicators.
5. Two condition indicators are calculated based on the extracted gear orders. These condition indicators are defined as ${ci}_{1}=\sum_{i}\left|sig_{o}\left[i\right]\right|$ and ${ci}_{2}=\sum_{i}\frac{\left|sig_{o}\left[i\right]\right|}{sig_{o}\left[hto\right]}$, where $sig_{o}$ is the synchronous average in the order domain, and hto represents the value at the hunting tooth order, equal to 1+35·99. This value is 1 for order zero and 35·99 for the first harmonic of the hunting tooth, corresponding to 35 planet gear teeth and 99 ring teeth. Lastly, i=1+99, 1+2·99, 1+3·99, etc.

The processed signal, i.e., the synchronous average of the hunting tooth, precisely comprises 99 complete revolutions of the cracked planet gear, as explained in Section 3. We anticipate the crack altering stiffness as a result of the crack’s “breathing”. Consequently, we expect to observe a periodic interference with approximately 99 revolutions in the processed hunting tooth synchronous average. The energy of this periodic interference manifests every 99 samples in the order domain, and consequently, ${ci}_{1}$ summarizes its energy. ${ci}_{2}$ normalizes ${ci}_{1}$ by the energy of the first hunting tooth order.

### 4.2. Estimation of the Severity and Remaining Time

**Figure 10:**
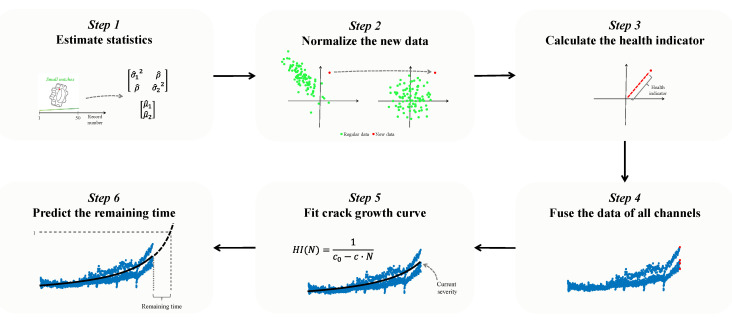
The data analysis for severity and remaining time estimation of the new digital twin.

The steps comprising the estimation of severity and remaining time are depicted in Figure 10. The main idea is to calculate a health indicator; when it reaches a value of 1, an alarm is raised, indicating that immediate maintenance action should be taken. When the health indicator reaches a value of 0.5, a warning is raised. The time the health indicator is between 0.5 and 1 is the time that the operator has to prepare for taking maintenance action [52]. It is assumed that the first 50 records represent the regular state before the propagation of the crack and can be used for learning the statistics of the regular state. The steps of this stage are as follows:

1. For each channel, the regular data are used to calculate the covariance matrix and the mean vector of the distribution of condition indicators.
2. For each new record in each channel, the extracted condition indicators are normalized according to the covariance matrix and mean vector calculated in Step 1.
3. A health indicator is calculated for each channel using the formula $HI=\frac{1}{A}\sqrt{{{\tilde{ci\;}}_{1}}^{2}+{{\tilde{ci\;}}_{2}}^{2}}$, where ${\tilde{ci\;}}_{1}$ and ${\tilde{ci\;}}_{2}$ are the normalized condition indicators from Step 2, and HI is the health indicator. A is a normalizing factor that is calculated as follows: based on the regular data, its value is estimated to ensure that when HI reaches the warning level of 0.5, the probability of a false alarm will be $10^{-6}$ [52]. This represents a statistically significant value, indicating a significant fault.
4. All health indicators of the four channels are fused together.
5. The current severity is calculated by fitting the trend of the four-channel health indicators to the crack propagation model based on Paris’ law [40,41] described in Section 2.3. It is assumed that $\frac{dHI}{dN}=C{\left(\sigma \cdot \alpha \cdot \sqrt{\pi }\right)}^{4}\cdot {HI}^{2}$, where the health indicator HI is approximately proportional to crack size, as will be demonstrated later, and N is the record number. The crack growth exponent is set to 4, which is a plausible standard value for steel [40]. By separating variables (HI and N) and integrating on both sides, the following solution to the differential equation is obtained: $-\frac{1}{HI}+c_{0}=C{\left(\sigma \cdot \alpha \cdot \sqrt{\pi }\right)}^{4}\cdot N,$ where $c_{0}$ is a constant. By rearranging the equation and denoting $c=C{\left(\sigma \cdot \alpha \cdot \sqrt{\pi }\right)}^{4}$, we obtain the final equation $HI(N)=\frac{1}{c_{0}-c\cdot N}$.

The fitting process consists of three steps: (1) $c_{0}$ is estimated using the regular data, assuming that c·N is negligible (in a real system, N can be considered 0 because there is no fault in the regular stage, and the Paris’ law is relevant just after the initiation of the fault). (2) A searching range for c is defined such that $c_{min}=\frac{1}{1000}\cdot \frac{1}{N_{min}\;}\left[-\frac{1}{min(HI)}+c_{0}\right]$ and $c_{max}=\frac{1}{N_{min}\;}\left[-\frac{1}{min(HI)}+c_{0}\right]$, where $N_{min}$ is the corresponding N value of min(HI). This value validates the largest HI value that can be fitted is in the searching range. (3) c is estimated by dividing the searching range into 1000 points on a logarithmic scale and finding the value of c in which the mean absolute error between the curve $HI(N)=\frac{1}{c_{0}-c\cdot N}$ and the calculated health indicators of Step 4 is minimal. The estimated severity is the HI of the current state.

6. The remaining time until immediate maintenance action should be taken is estimated based on the estimated curve, indicating how many records it will take for the health indicator to reach a value of 1.

### 4.3. Demonstration on the HUMS 2023 Benchmark Dataset

**Figure 11:**
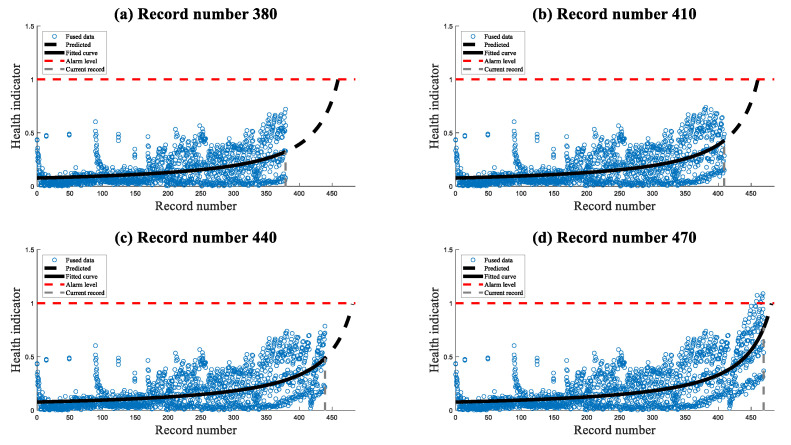
Four examples of different current processed records of the digital twin severity estimation and prediction trends.

The new digital twin was applied to the measured data from the HUMS 2023 benchmark dataset described in Section 3. This analysis revealed two key results: a comparison between the online estimated fault severity and the final estimated fault severity when all data were available, and an estimation of the remaining time until immediate maintenance action should be taken. The examples of the digital twin severity estimation and prediction trends are presented in Figure 11.

The challenge of predicting the propagation of a gear root crack without examples from other systems remains open. This presents a type of zero-fault-shot learning, necessitating the estimation of the severity and the time remaining until an immediate maintenance action should be taken without prior exposure to faulty examples. The introduction of digital twinning offers a means to tackle this problem. Given its status as an open problem, there are presently no alternative algorithms available for comparison with the digital twin.

In the HUMS 2023 dataset, each record is obtained after angular resampling and synchronous averaging. Therefore, the first two steps of the signal processing stage do not need to be utilized.

**Figure 12:**
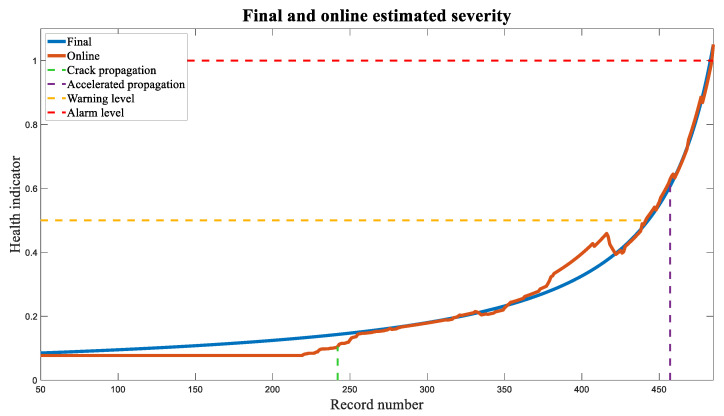
Comparison between the online estimated severity and the final estimated severity when the health indicator reaches 1. The times of crack propagation initiation and the beginning of accelerated propagation were added to the graph.

The severity at each point was estimated using the algorithm described in Section 4.2. The results are presented in Figure 12. The comparison was made until the health indicator reached 1 in Record 485, at which point an alarm was triggered, and the use of the physical asset should be immediately stopped. The online trend is not a perfectly fitted curve type like the final curve because the fitted curve in Step 5 in Section 4.2 varies as new records arrive.

As shown in Figure 12, the online and final trends exhibit good agreement. Additionally, two lines were added to indicate the estimated start of the crack propagation and accelerated propagation based on fractography analysis. It is evident that the online estimated severity of the digital twin closely aligns with these two points. Near the onset of crack propagation, at Record 252, online severity also starts to increase. At Record 457, between the warning level at 0.5 and the alarm level at 1, accelerated propagation begins, indicating the need for immediate maintenance action several records in advance. In fact, the digital twin raises an alarm at Record 485.

**Figure 13:**
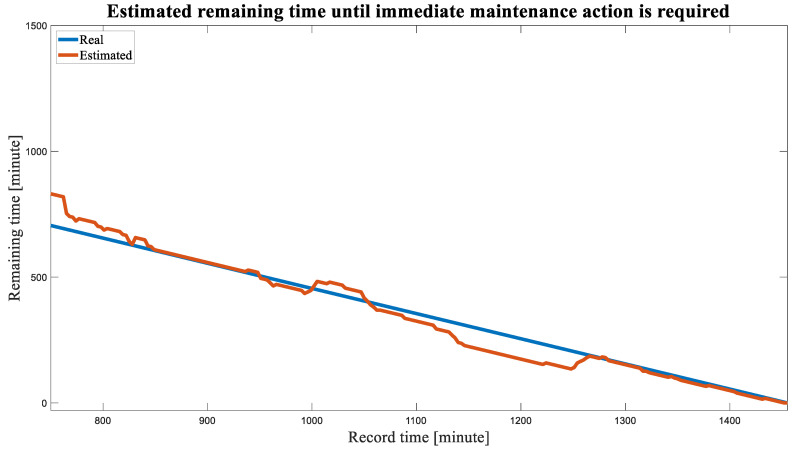
Comparing the real and estimated time remaining until immediate maintenance action is required when the health indicator reaches the value of 1.

A comparison of the estimated remaining time until immediate maintenance action should be taken is presented in Figure 13, where the axes are represented in minutes based on the 3 min intervals between consecutive records. As shown in the figure, there is a strong agreement between the actual and estimated remaining time.

These online outputs of the digital twin, aka fault severity and remaining time estimations, enable making significant maintenance decisions, such as determining when immediate maintenance action should be taken and how many flight hours remain before this action becomes necessary. This information can be highly valuable in various practical scenarios, for instance, identifying which helicopter should be the first to undergo maintenance actions and determining their priority. Moreover, helicopters with a short remaining time until maintenance can be excluded from tasks that may be time-consuming and could potentially result in an abrupt grounding of the helicopter.

## 5. Summary

Digital twins play a pivotal role in Industry 4.0. This paper demonstrates how digital twinning can assist in one of the critical tasks of condition-based maintenance by assessing the current fault severity and predicting when immediate maintenance action should be taken. The new digital twin involves signal processing, the assessment of the severity of the current state, and predicting crack evolution using the Paris law for crack propagation. The capabilities of this digital twin were demonstrated on the HUMS 2023 benchmark dataset, including both the severity assessment and the prediction of the remaining time until immediate maintenance is necessary. This study demonstrates the ability of digital twinning to address one of the core problems of condition-based maintenance, which is to estimate fault severity and remaining time until maintenance under zero-fault-shot learning, where fault data are not provided during the training phase.

Digital twinning holds significant potential in the field of condition-based maintenance, as highlighted in this article. In the future, the use of digital twinning can be expanded to cover a broader range of components and faults, providing a more accurate representation of various machine parts. For example, a digital twin can be developed for complex machinery, where it will help monitor faults in the bearing, shaft, and gear by classifying the fault type, estimating the severity, and predicting the remaining time until maintenance.

## Data Availability

HUMS 2023 dataset is available via Ref. [26].
